# Plasma mitochondrial DNA and metabolomic alterations in severe critical illness

**DOI:** 10.1186/s13054-018-2275-7

**Published:** 2018-12-29

**Authors:** Pär I. Johansson, Kiichi Nakahira, Angela J. Rogers, Michael J. McGeachie, Rebecca M. Baron, Laura E. Fredenburgh, John Harrington, Augustine M. K. Choi, Kenneth B. Christopher

**Affiliations:** 1 Department of Clinical Immunology, Copenhagen University Hospital, Copenhagen, Denmark; 2 Division of Pulmonary and Critical Care Medicine, Department of Medicine, Weill Cornell Medicine, New York, NY USA; 3 Pulmonary & Critical Care Medicine, Stanford University Medical Center, Stanford, CA USA; 4 Channing Division of Network Medicine, Department of Medicine, Brigham and Women’s Hospital, Boston, MA USA; 5 Pulmonary and Critical Care Division, Department of Medicine, Brigham and Women’s Hospital, Boston, MA USA; 6 Division of Pulmonary and Critical Care Medicine, Department of Medicine, New York Presbyterian-Weill Cornell Medical Center, Weill Cornell Medicine, New York, NY USA; 7 Department of Medicine, New York-Presbyterian Hospital, New York, NY USA; 8 Renal Division, Department of Medicine, Brigham and Women’s Hospital, 75 Francis Street, MRB 418, Boston, MA 02115 USA

**Keywords:** Mitochondrial DNA, Metabolite, Metabolomics, Homeostasis, Critical illness, Acylcarnitine, Glycerophosphocholine

## Abstract

**Background:**

Cell-free plasma mitochondrial DNA (mtDNA) levels are associated with endothelial dysfunction and differential outcomes in critical illness. A substantial alteration in metabolic homeostasis is commonly observed in severe critical illness. We hypothesized that metabolic profiles significantly differ between critically ill patients relative to their level of plasma mtDNA.

**Methods:**

We performed a metabolomic study with biorepository plasma samples collected from 73 adults with systemic inflammatory response syndrome or sepsis at a single academic medical center. Patients were treated in a 20-bed medical ICU between 2008 and 2010. To identify key metabolites and metabolic pathways related to plasma NADH dehydrogenase 1 (ND1) mtDNA levels in critical illness, we first generated metabolomic data using gas and liquid chromatography-mass spectroscopy. We performed fold change analysis and volcano plot visualization based on false discovery rate-adjusted *p* values to evaluate the distribution of individual metabolite concentrations relative to ND1 mtDNA levels. We followed this by performing orthogonal partial least squares discriminant analysis to identify individual metabolites that discriminated ND1 mtDNA groups. We then interrogated the entire metabolomic profile using pathway overrepresentation analysis to identify groups of metabolite pathways that were different relative to ND1 mtDNA levels.

**Results:**

Metabolomic profiles significantly differed in critically ill patients with ND1 mtDNA levels ≥ 3200 copies/μl plasma relative to those with an ND1 mtDNA level < 3200 copies/μl plasma. Several analytical strategies showed that patients with ND1 mtDNA levels ≥ 3200 copies/μl plasma had significant decreases in glycerophosphocholines and increases in short-chain acylcarnitines.

**Conclusions:**

Differential metabolic profiles during critical illness are associated with cell-free plasma ND1 mtDNA levels that are indicative of cell damage. Elevated plasma ND1 mtDNA levels are associated with decreases in glycerophosphocholines and increases in short-chain acylcarnitines that reflect phospholipid metabolism dysregulation and decreased mitochondrial function, respectively.

**Electronic supplementary material:**

The online version of this article (10.1186/s13054-018-2275-7) contains supplementary material, which is available to authorized users.

## Background

Mitochondrial function is a major determinant of outcome in critical illness. Circulating mitochondrial damage-associated molecular patterns (DAMPs), such as cell-free mitochondrial DNA (mtDNA), contain unmethylated CpG and formylated peptides that activate immune responses through Toll-like receptor 9 and formyl peptide receptors, respectively [1–4].

Plasma mtDNA is measurable in critically ill patients, with increasing levels associated with sepsis, sepsis disease severity, and mortality [5–7]. The primary factors in the extracellular release of mtDNA are cell stress and necrosis [3]. Experimental data published in abstract form showed an increase in extracellular mtDNA by induction of endothelial cell necroptosis following transfusion [8]. Mitochondria-related DAMPs from damaged, dying, or dead cells appear to be important for the early systemic endothelial response to sepsis [9]. mtDNA is shown to increase endothelial cell permeability, either directly or through interactions with endothelial cells and polymorphonuclear leukocytes [9]. These findings suggest that plasma mtDNA levels could reflect the level of injury and may also reflect the level of dysfunction or damage that mitochondria undergo in response to physiologic stress [10].

Because metabolic homeostasis is often disrupted in critical illness, substantial alterations of several intrinsic pathways can be expected in septic patients [11]. To date, a number of metabolomic studies have been published in experimental sepsis models [12], pediatric sepsis [13], and adult critically ill patients [14–17]. Circulating metabolic signatures showing alteration in fatty acids, lipids, and tryptophan pathways are prominent in cohorts of septic patients [14–17].

Existing data support that mtDNA is related to the activation of inflammation and organ dysfunction [18]. However, there is limited understanding of the metabolic alterations associated with elevated mtDNA levels in critical illness. Therefore, we analyzed metabolite profiles with regard to NADH dehydrogenase 1 (ND1) mtDNA levels in a prospective study of adult patients with systemic inflammatory response syndrome (SIRS) and sepsis [19]. The ND1 protein is a subunit of NADH dehydrogenase found in the inner membrane of mitochondria [20]. We hypothesized that the metabolomic profile of critically ill patients near intensive care unit (ICU) admission differs in patients with elevated ND1 mtDNA levels and that this difference can illuminate important biologic pathways related to the response to mitochondrial DAMPs.

## Methods

### Study design and patients

The Registry of Critical Illness (RoCI) is a registry of adult medical ICU patients based at the Brigham and Women’s Hospital (Boston, MA, USA), created to record patient data and store samples for plasma, RNA/DNA analysis, and protein isolation. The protocol for patient recruitment has been previously described at length [19]. Between September 2008 and May 2010, 90 medical ICU patients had metabolic profiling performed; of these, 29 patients satisfied SIRS criteria, 30 patients satisfied criteria for sepsis, and 31 patients satisfied criteria for sepsis and acute respiratory distress syndrome [15]. Cases were not selected with regard to risk of death or any known metabolic feature. We conducted a subanalysis involving 73 RoCI patients who had been selected for metabolic profiling [15] and in whom cell-free plasma ND1 mtDNA levels were determined in a prior study of mtDNA [5].

### Exposure of interest and comorbidities

The exposure of interest was cell-free circulating plasma ND1 mtDNA assessed by measuring copy number of the *ND1* gene using qRT-PCR [5]. ND1 mtDNA level was assessed as a binary variable (ND1 mtDNA ≥ 3200 copies/μl plasma vs. ND1 mtDNA level < 3200 copies/μl plasma). The cut point of ND1 mtDNA level of 3200 copies/μl plasma was determined in our prior study to maximize the AUC for the prediction of 28-day mortality [5]. The preparation and quantification of plasma ND1 mtDNA is outlined in Additional file 1. Demographic and physiologic data were collected from the clinical record as described previously [19]. In addition to data collected by the RoCI, supplemental data on all patients were compiled through a hospital-based computerized data registry [21] as outlined in Additional file 1.

Metabolomic profiling identified 411 metabolites for the complete RoCI cohort (*N* = 90 plasma samples within 72 h of ICU admission) using Metabolon, Inc. (Morrisville, NC, USA) [15]. Gas and liquid chromatography mass spectroscopy (GC-MS, LC-MS) were performed as described previously [22, 23]. We removed metabolites with the lowest IQR of variability in the RoCI data, leaving 308 metabolites. This strategy is commonly used to reduce baseline noise by removing constant or very weak variables [24, 25]. All metabolite concentrations were log_2_-transformed to normalize the data that were used for all of the models and all of the metabolite data analyses. Details on metabolomic sample processing have been described at length previously and are outlined in Additional file 1 [15].

We used MetaboAnalyst 4.0 software (www.metaboanalyst.ca) to identify key metabolism alterations related to ND1 mtDNA level [26]. Univariate tests, including fold change analysis and volcano plot visualization based on false discovery rate (FDR)-adjusted *p* values, were performed to evaluate the distribution of individual metabolite concentrations in individuals with elevated ND1 mtDNA levels (≥ 3200 copies/μl plasma) relative to those with ND1 mtDNA levels < 3200 copies/μl plasma [5]. Cross-sectional correlations were calculated using Pearson’s product-moment correlation (*r*) between metabolites and ND1 mtDNA levels. For data visualization purposes, a bipartite graph was generated of metabolites that were significantly changed (increased or decreased) with elevated ND1 mtDNA level (≥ 3200 copies/μl plasma). Significant features were further identified by significance analysis of microarrays (SAM) [27], in which the FDR was determined by running multiple tests on high-dimensional data that distinguish between patients with ND1 mtDNA ≥ 3200 copies/μl plasma relative to those with ND1 mtDNA < 3200 copies/μl plasma, with a *q* value (upper limit of FDR) < 0.01 considered to be significant.

We performed logistic regression with ND1 mtDNA ≥ 3200 copies/μl plasma as the exposure and 28-day mortality as the outcome, after adjustment for Acute Physiology and Chronic Health Evaluation II (APACHE II) score and sepsis. Linear regression was performed with ND1 mtDNA ≥ 3200 copies/μl plasma as the exposure and acylcarnitine metabolites as the outcome after adjustment for age, sex, race, and APACHE II score. Linear regression was also performed with ND1 mtDNA copies/μl plasma as the exposure and individual metabolites as the outcome adjusted for age, sex, race, and APACHE II score. STATA 14.1/MP software (StataCorp, College Station, TX, USA) was used for all regression analyses.

Orthogonal partial least squares discriminant analysis (OPLS-DA), a supervised method, was used to select variables representing the greatest contribution to classification of the ND1 mtDNA groups [28]. The quality of the multivariate model developed was described by R2 and Q2, which corresponded to the model’s goodness of fit and predictive performance, respectively. Permutation testing was performed to validate the OPLS-DA model [29, 30]. Sevenfold cross-validation analysis of variance (CV-ANOVA) was applied to determine OPLS-DA model significance [30]. Variables that contributed the most to ND1 mtDNA group recognition were identified with SIMCA (Umetrics, Umeå, Sweden) using variable importance in the projection (VIP) scores. VIP scores > 1 are considered to be important for the explanatory/predictive ability of an OPLS-DA model [31]. We used an S-plot to visualize the variable influence between ND1 mtDNA groups in the OPLS-DA model by combining the contribution/covariance and reliability/correlation loading profiles [28]. A correlation coefficient of ± 0.410 was adopted as a cutoff value to select the variables that are most correlated with the OPLS-DA discriminant scores.

For pathway overrepresentation analysis of case-control metabolite data, metabolomic pathway analysis (MetPA) [32] was used. MetPA was used to evaluate a list of the 308 metabolites and their log-normalized concentration data in the 73 samples by comparing patients with ND1 mtDNA ≥ 3200 copies/μl plasma (cases) with those with ND1 mtDNA < 3200 copies/μl plasma (controls). Metabolite set enrichment analysis was performed by mapping the metabolite data onto the Human Metabolome Database (HMDB) [33]. Metabolites were evaluated for pathway enrichment using the “*Homo sapiens*” library with the default parameters (“Global Test” and “Relative Betweenness Centrality”) specified as the algorithms for pathway enrichment and topological analysis, respectively. The resulting metabolic networks were represented as directed graphs, and centrality measures of a metabolite within a given network were then applied to estimate the relative importance of that metabolite in the network. Fisher’s exact test *p* values were adjusted for multiple testing using the Holm-Bonferroni method [34].

## Results

Table 1 shows demographic characteristics of the study cohort. Most patients were male (53%) and white (78%). The mean (SD) age at ICU admission was 54 (15) years. The mean (SD) APACHE II score was 26 (10), and 70% of the cohort patients were diagnosed with sepsis. The 28-day mortality within the cohort was 37%. Significant differences existed in patients with and without ND1 mtDNA ≥ 3200 copies/μl plasma in regard to APACHE II, sepsis, and 28-day mortality (Table 1). Patients with ND1 mtDNA ≥ 3200 copies/μl plasma had a sixfold higher odds of 28-day mortality following adjustment for APACHE II and sepsis compared with patients with ND1 mtDNA < 3200 copies/μl plasma (OR, 6.4; 95% CI, 1.8–22.9; *p* = 0.004), similar to what was reported in the parent ND1 mtDNA study [5].

**Table 1:** Patient characteristics

Characteristics	ND1 mtDNA copies/μl plasma		*p* Value
	< 3200	≥ 3200	
No. of patients	35	38	
Age, years, mean (SD)	53.6 (16.2)	55.2 (13.7)	0.64
Male sex, *n* (%)	19 (48.7)	20 (51.3)	0.89
White race, *n* (%)	27 (77.1)	30 (79.0)	0.92
APACHE II score, mean (SD)	21.8 (9.8)	29.2 (9.3)	0.0015
Sepsis, *n* (%)	17 (33.3)	34 (66.7)	< 0.001
28-Day mortality, *n* (%)	5 (14.3)	22 (57.9)	< 0.001

### Primary outcome

**Fig. 1:**
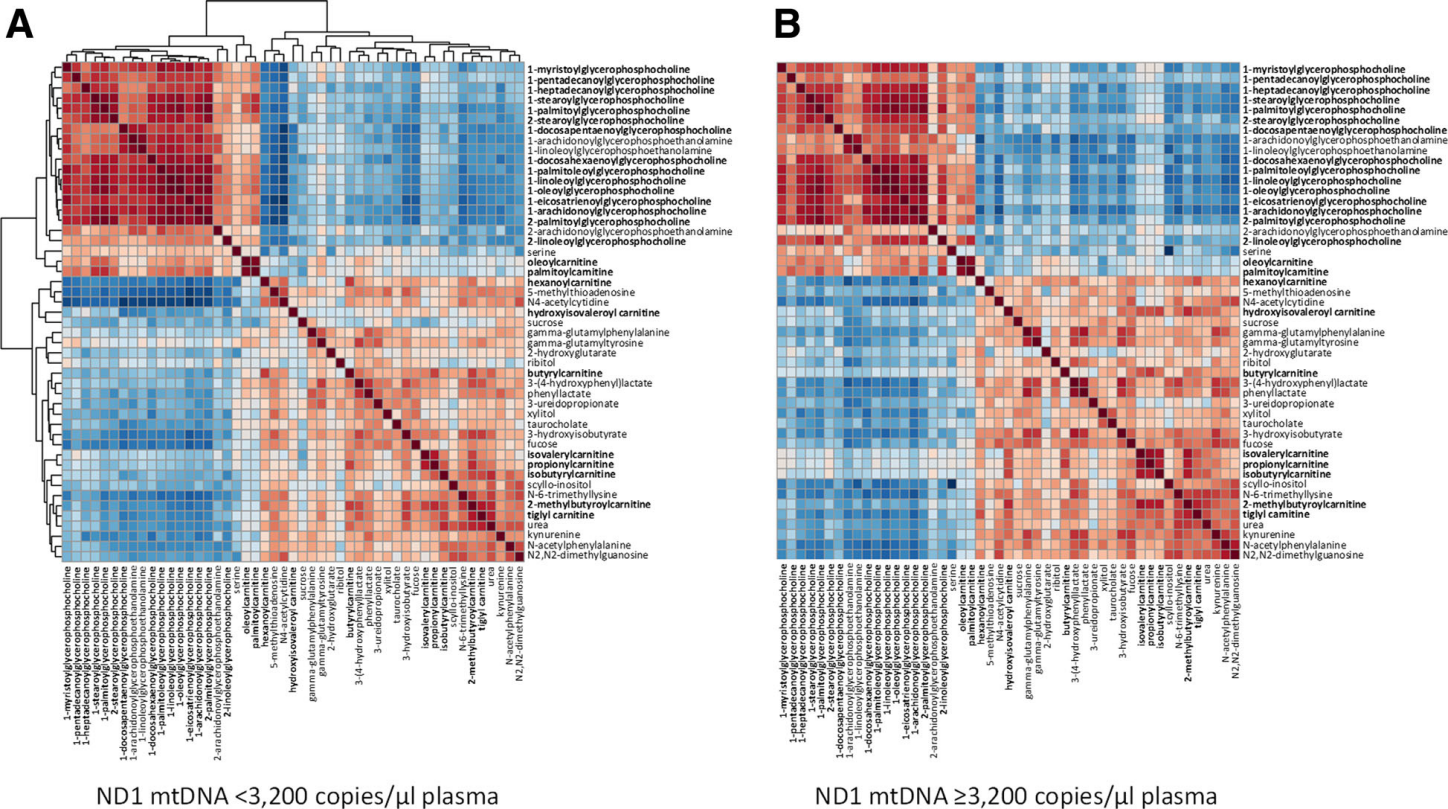
Hierarchical clustering of correlated metabolites relative to NADH dehydrogenase 1 (ND1) mitochondrial DNA (mtDNA) group level. Correlation matrix of the 49 major differential metabolites from Additional file 2: Table S1. Cross-sectional correlation colors represent Pearson correlation coefficients of log-transformed metabolites by ND1 mtDNA levels. **a** ND1 mtDNA < 3200 copies/μl plasma. **b** ND1 mtDNA ≥ 3200 copies/μl plasma. Glycerophosphocholine and short-chain acylcarnitines are marked in *bold*. *Red* and *blue* indicate positive and negative correlations, respectively. Metabolites with marked differences in the degree of intercorrelation between ND1 mtDNA groups were the glycerophosphocholines (1-stearoylglycerophosphocholine, 2-stearoylglycerophosphocholine, 1-palmitoylglycerophosphocholine) and the short-chain acylcarnitines (isovalerylcarnitine, propionylcarnitine and isobutyrylcarnitine). This analysis allows for the identification and clustering of metabolite modules of related function in patients with low mtDNA and how those correlations are altered in patients with high mtDNA

Metabolomic profiles significantly differed in critically ill patients with ND1 mtDNA ≥ 3200 copies/μl plasma relative to those with ND1 mtDNA < 3200 copies/μl plasma (Additional file 2 Table S1). To illustrate metabolite modules that are potentially biosynthetically linked, a correlation matrix derived from the log-transformed metabolite concentration is shown in Fig. 1. A large cluster of correlated glycerophosphocholine metabolites is present in patients with ND1 mtDNA < 3200 copies/μl plasma and present but less correlated in those with ND1 mtDNA ≥ 3200 copies/μl plasma (Fig. 1a, b). A small cluster of short-chain acylcarnitines, including propionylcarnitine (C3), isobutyrylcarnitine (C4), and isovalerylcarnitine (C5), are accentuated in patients with ND1 mtDNA ≥ 3200 copies/μl plasma (Fig. 1b).

**Fig. 2:**
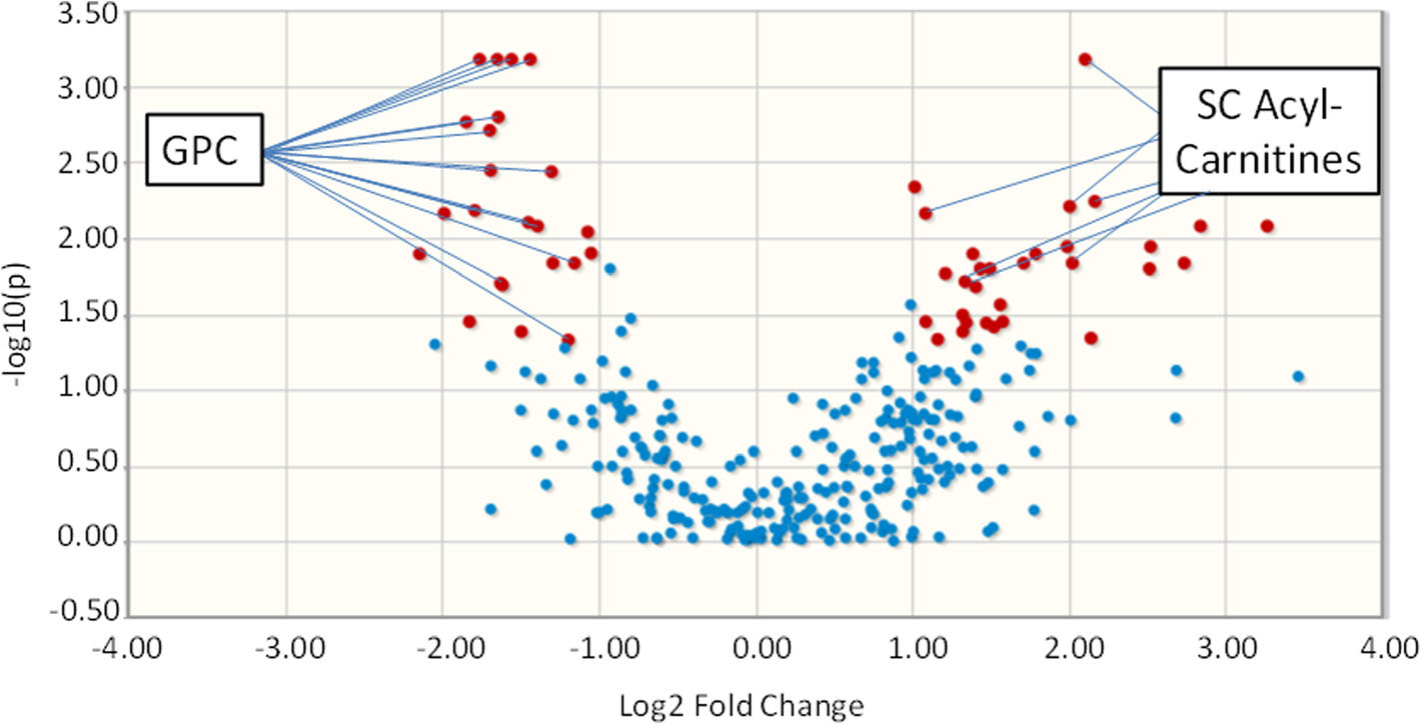
Volcano plot of differential metabolites. In the volcano plot, differential metabolites (*red*) and nondifferential substances (*blue*) were determined under the conditions of fold change ≥ 2 and false discovery rate-adjusted *p* value threshold ≤ 0.05. The *p* values are transformed by −log_10_ so that the more significantly different metabolites (with smaller *p* values) are higher on the *y*-axis. The fold change is log-transformed so that negative values represent a decrease in metabolite levels and positive values represent an increase in metabolite levels. *Red* and *blue* indicate notable and nonnotable metabolites, respectively. *GPC* Glycerophosphocholine, *SC* Short-chain acylcarnitine

**Fig. 3:**
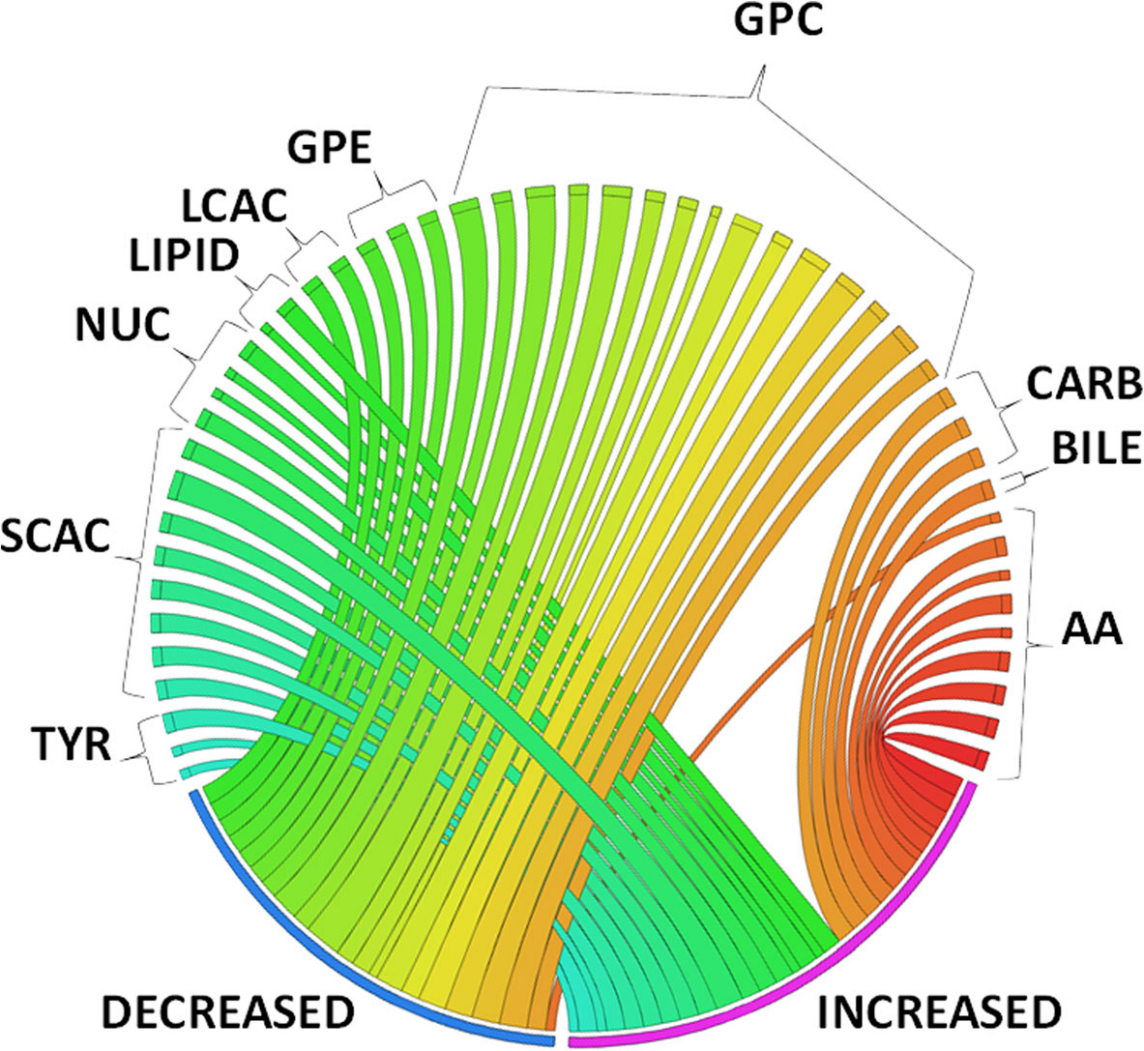
Circos plot of differential metabolites. Bipartite graph of metabolites significantly changed (increased or decreased) with NADH dehydrogenase 1 (ND1) mitochondrial DNA (mtDNA) ≥ 3200 copies/μl plasma. Graph connects response to ND1 mtDNA ≥ 3200 copies/μl plasma with individual increased or decreased metabolites. Width of curves indicates strength of the significance (−log_10_(*p*) value). *AA* Amino acid metabolites, *BILE* Bile acids, *CARB* Carbohydrates, *GPC* Glycerophosphocholines, *GPE* Glycerophosphoethanolamines, *LCAC* Long-chain acylcarnitines, *LIPID* Lipid metabolites, *NUC* Nucleotide metabolism, *SCAC* Short-chain acylcarnitines, *TYR* Tyrosine metabolites

The volcano plot in Fig. 2 graphically shows the data presented in Additional file 2: Table S1, highlighting the relationship between the FDR-adjusted *p* values and the magnitude of the fold change difference in metabolite concentrations with respect to ND1 mtDNA levels. Notable is the significant increase of short-chain acylcarnitines (C4–C6) and the decrease in both glycerophosphocholines and long-chain acylcarnitines (C16–C18) in those with elevated ND1 mtDNA level (≥ 3200 copies/μl plasma) (Fig. 2). A bipartite graph of notable volcano plot metabolites significantly changed (increased or decreased) with ND1 mtDNA ≥ 3200 copies/μl plasma illustrates the prominence of increase of short-chain acylcarnitines and the decrease of both glycerophosphocholines and long-chain acylcarnitines (Fig. 3). Adjusted linear regression of ND1 mtDNA as a continuous exposure with individual metabolites as the outcome showed a similar prominence of increase of short-chain acylcarnitines and the decrease of glycerophosphocholines (Additional file 2: Table S2). The observed acylcarnitine ester metabolite pattern of notable volcano plot metabolites relative to ND1 mtDNA level was maintained following multivariable linear regression (Additional file 3: Figure S1). SAM [27] further identified metabolite features in patients with ND1 mtDNA ≥ 3200 copies/μl plasma relative to those with ND1 mtDNA < 3200 copies/μl plasma (Additional file 2: Table S3). The SAM included significant increases in the short-chain acylcarnitines and decreases in both glycerophosphocholines and long-chain acylcarnitines in patients with ND1 mtDNA ≥ 3200 copies/μl plasma.

For the OPLS-DA, a supervised multivariate analysis, the robustness and reliability of the model were marginal (Table 2). In addition to the R2 and Q2 metrics, the response permutation test (with *n* = 200) was used to validate the predictive capability of the computed OPLS-DA models [35]. Though the OPLS-DA model had marginal predictability, the permutation test confirmed the stability and robustness of the model (Q2 intercept, − 0.26; *p* ≤ 0.05) with a negative permutation Q2 intercept indicating model validity [29, 30] (Table 2). The cross-validation procedure showed that the two ND1 mtDNA groups were significantly separated (CV-ANOVA *p* value = 0.00013). By combining the VIP values in the loadings plot, 34 metabolites with VIP > 1 were selected as differentially accumulated metabolites (Additional file 2: Table S4). Metabolites with VIP > 1 included short-chain acylcarnitines and glycerophosphocholines. At the correlation coefficient cut point of ±0.410, the S-plot identified differential metabolites between ND1 mtDNA groups. These differential metabolites included increases in the short-chain acylcarnitines and decreases in both glycerophosphocholines and long-chain acylcarnitines in patients with ND1 mtDNA ≥ 3200 copies/μl plasma (Additional file 2: Table S5).

**Table 2:** Cross-validation and permutation

OPLS-DA			Permutation (*n* = 200)	
R2X	R2Y	Q2	R2 intercept (*x*-axis, *y*-axis)	Q2 intercept (*x*-axis, *y*-axis)
0.190	0.333	0.226	(0.00, 0.283)	(0.00, − 0.264)

We next sought to identify differential biologically meaningful metabolite pathways in the cohort with regard to ND1 mtDNA status. Two hundred thirty-four of the 308 metabolites mapped to the HMDB. An HMDB-pathway match was absent for 46 metabolites, and a lack of an HMDB assignment was present for 28 metabolites. The acylcarnitine metabolites did not map to the HMDB and thus were not included in the MetPA output. The MetPA-identified metabolites most significantly enriched in patients with ND1 mtDNA ≥ 3200 copies/μl plasma were related to glycerophospholipid metabolism (FDR-adjusted *p* < 0.001; pathway impact score, 0.138) and tryptophan metabolism (FDR-adjusted *p* = 0.018; pathway impact score, 0.249) (Table 3).

**Table 3:** Metabolomics pathway analysis

Pathway name	Total no. of metabolites	No. of overlapping metabolites	Unadjusted *p* value	FDR-adjusted *p* value	Pathway impact score
Glycerophospholipid metabolism	39	6	0.00003	0.00090	0.139
Pyrimidine metabolism	60	6	0.00018	0.00351	0.028
Galactose metabolism	41	6	0.00086	0.01146	0.020
Lysine degradation	47	3	0.00149	0.01438	0.024
Starch and sucrose metabolism	50	6	0.00240	0.01739	0.119
Tryptophan metabolism	79	6	0.00279	0.01797	0.249

*FDR* False discovery rate

## Discussion

In the present study, our goal was to determine if metabolite signatures in critically ill patients would be distinct relative to cell-free plasma ND1 mtDNA levels. Using high-resolution metabolomics, we demonstrated substantial differences in glycerophospholipid and acylcarnitine family member metabolism based on the level of ND1 mtDNA liberated in the plasma. Specifically, patients with high levels of plasma ND1 mtDNA, indicative of cellular damage, have very low levels of multiple glycerophosphocholine esters and increased levels of several short-chain acylcarnitines.

In cohorts of septic patients, alterations in circulating kynurenines, fatty acids, lysophosphatidylcholines, and/or carnitine esters [14–17] indicate a substantial disturbance in energy and lipid homeostasis that occurs with increasing severity of illness. Large decreases in glycerophosphocholines are demonstrated in patients with experimental infection with *Bacillus anthracis* spores [36], bacteremia [37], and sepsis [17] and appear to correlate with sepsis mortality [38]. Glycerophosphocholines are water-soluble compounds formed in the breakdown of phosphatidylcholine via phospholipase A1 and phospholipase A2 activities, and they are degraded by glycerophosphodiester phosphodiesterases [39]. Glycerophosphocholines are essential components of biological membranes that modulate membrane trafficking and control cell viability [39]. Glycerophosphocholines function in glycerophospholipid, prostaglandin, and leukotriene metabolism; are important in energy storage, signal transduction, and membrane physiology; provide mitochondrial support; and are neutrophil-activating factors [36].

The observed substantial decreases in glycerophosphocholines during sepsis may be related to increased glycerophosphocholine hydrolysis [37]. Circulating phospholipase A2 activity is found in sepsis [40]. Additionally, endothelial cells secrete the phospholipase endothelial lipase (EL) involved in phospholipid homeostasis [41, 42]. EL is produced by macrophages in addition to the endothelium in response to plasma inflammation markers [43]. In human experimental models of low-dose endotoxemia, significant augmentation of plasma EL concentrations has been shown [44]. The combination of circulating phospholipase A2 and EL activity may be responsible for the low glycerophosphocholine metabolites observed in our study.

Lipidomic alterations are prominent in sepsis and critically ill patients [45–48]. We have shown that carnitine esters, important for immune response to pathogens [49], are the most pronounced metabolites that differed between sepsis nonsurvivors and survivors [17]. Alterations of acylcarnitines are found in studies of severe sepsis/septic shock [45], the prediction of death in sepsis [17], and an integrative omics study in primates that was validated also in human patient cohorts [50].

In critical illness, metabolic pathways are altered to preferentially catabolize fatty acids and amino acids. Substantive literature demonstrates that an early indicator of critical illness outcomes is mitochondrial biogenesis [51–54]. Elevated short-chain acylcarnitines found in plasma are due to incomplete mitochondrial fatty acid β-oxidation downstream of carnitine palmitoyltransferase I and are suggestive of impaired mitochondrial function [55–57]. The increase in plasma short-chain acylcarnitines with elevated ND1 mtDNA in our study may reflect less efficient fatty acid β-oxidation, potentially thorough worsening of mitochondrial bioenergetics.

Accelerated tryptophan catabolism along the kynurenine pathway occurs with sepsis. The enzyme responsible for kynurenine production is upregulated by bacterial products and is critically involved in CD4^+^ and CD8^+^ effector T-cell suppression as well as in generation and activation of regulatory T cells [58, 59]. We and others have found that modulation of kynurenine is associated with 28-day mortality in sepsis [14, 15]. Increased production of kynurenine has been proposed to contribute to hypotension in sepsis [60] and has been associated with dysregulated immune response and impaired microvascular reactivity [61].

Strengths of the present study include using cell-free plasma for ND1 mtDNA measurement. Because platelets secrete their mitochondria following activation during inflammation and sepsis, they may serve as a source of extracellular mtDNA [62]. Using cell-free plasma allowed us to draw the inference that the source of ND1 mtDNA is more likely the endothelium. Further, we employed several types of statistical procedures, and data visualization processes were used to identify differential metabolites, including Student’s *t* test [63], Pearson correlation, volcano plot, bipartite graph, SAM [27], OPLS-DA, and MetPA [32].

The present study is not without potential limitations. Metabolites were measured early in the ICU course of severe critical illness, from a relatively small number of patients, at a single time point, and from a single biofluid (plasma). Our assumption that plasma is an integrative biofluid may not account for tissue- or organ-specific metabolism. Our observational study included patients who were critically ill for various reasons, creating a heterogeneous study sample with high severity of illness. Further, selection bias may be present because we analyzed only a subset of patients of the RoCI cohort who had ND1 mtDNA determined. We are unable to account for the impact of race on metabolic profiles because our cohort was mostly white. Because our study was performed on a convenience sample and not replicated in other cohorts, our results may not be generalizable to all critically ill patients. Our bioinformatics approaches, while robust, are not without risk of introducing sources of bias. Single–time point metabolomics provides important information but does not capture the dynamic changes over time [64]. We were not able to determine the stability of metabolites over storage time [65]. Although OPLS-DA is well-suited for metabolomic data with much larger numbers of predictors than observations and multicollinearity, it is prone to overfitting; however, permutation testing indicated a low likelihood of seeing results this strong by chance (*p* ≤ 0.05) [29, 30]. Like in our study, mapping the metabolite data onto the HMDB [33] does not always result in HMDB number assignment to each metabolite. Finally, we cannot fully account for potential confounding, reverse causation, and the lack of a randomly distributed exposure [66].

## Conclusions

In summary, elevated levels of cell-free plasma ND1 mtDNA are associated with differential metabolic profiles in early severe critical illness. Glycerophospholipids, which are important in phospholipid metabolism and mitochondrial support, are significantly depressed in patients with high plasma ND1 mtDNA. Short-chain acylcarnitines indicative of mitochondrial dysfunction are increased with high plasma ND1 mtDNA. These data, although observational, provide an important window into the metabolite signatures of cell stress and necrosis in response to sepsis.

## Additional files

Additional file 1: Supplemental Methods. Additional methods. (DOC 115 kb)

Additional file 2: **Table S1.** Metabolites identified via volcano plot with significant differences in patients with ND1 mtDNA ≥ 3200 copies/μl plasma relative to those with ND1 mtDNA < 3200 copies/μl plasma. **Table S2.** Linear regression of significant metabolites relative to every 100 ND1 mtDNA copies/μl plasma analyzed as a continuous exposure. **Table S3.** Metabolites identified by SAM with significant differences in patients with ND1 mtDNA ≥ 3200 copies/μl plasma relative to those with ND1 mtDNA < 3200 copies/μl plasma. (DOCX 33 kb)

Additional file 3: **Figure S1.** Acylcarnitine association plot. Logistic regression results of 13 acylcarnitine esters in 73 patients. Each dot is a single acylcarnitine ester detected. Color indicates the relative acylcarnitine ester association with ND1 mtDNA ≥ 3200 copies/μl plasma (red increased, blue decreased) after adjustment for age, sex, race, malignancy, and APACHE II score. *y*-Axis is (−log_10_(*p*) value). *x*-Axis is acylcarnitine chain length C3 to C18. (DOCX 38 kb)

## Abbreviations

APACHE II: Acute Physiology and Chronic Health Evaluation II
CV-ANOVA: Cross-validation analysis of variance
DAMPs: Damage-associated molecular patterns
EL: Endothelial lipase
FDR: False discovery rate
HMDB: Human Metabolome Database
ICU: Intensive care unit
MetPA: Metabolomic pathway analysis
mtDNA: Mitochondrial DNA
ND1: NADH dehydrogenase 1
OPLS-DA: Orthogonal partial least squares discriminant analysis
RoCI: Registry of Critical Illness
SAM: Significance analysis of microarrays
SIRS: Systemic inflammatory response syndrome
VIP: Variable importance in the projection
