# Supplementary material for: Plasma mitochondrial DNA and metabolomic alterations in severe critical illness
Source: Crit Care. 2018 Dec 29;22:360. doi: 10.1186/s13054-018-2275-7 (PMC6310975; doi:10.1186/s13054-018-2275-7)
Supplement: Supplementary file 1 — Supplemental Methods. Additional methods. (DOC 115 kb) [file 13054_2018_2275_MOESM1_ESM.doc]

**Supplemental Methods**

The Brigham and Women’s Hospital Registry of Critical Illness (RoCI) is approved by the Partners IRB committee (2008-P-000495). The protocol for recruitment for RoCI has been published in detail elsewhere (1). Briefly, adult patient (over age 18) who is admitted to the BWH Medical Intensive Care Unit is eligible for inclusion in the RoCI within 72 hours of presentation, unless certain exclusion criteria are met (unable to provide consent due to cognitive dysfunction or no appropriate health care proxy, prior refusal, admission purely for comfort care, Jehovah’s Witness status, or a baseline hemoglobin <8 g/dL or hemoglobin <9 g/dL with either admission for active bleeding or with acute ischemia). Plasma is obtained on Days 1, 3, and 7 of enrollment. Extensive phenotypic data (including age, gender, key comorbidities, and APACHE II score), laboratory and radiologic data are recorded for all subjects. Classification of Systemic Inflammatory Response (SIRS), Sepsis, and ARDS is determined by a consensus panel of ICU physicians using the current disease classification (2, 3).

We utilized metabolomic data that was produced by Rogers et.al. (4) from the Registry of Critical Illness (RoCI) cohort described by Dolinay et.al. (1). Among 225 subjects described by Dolinay et.al. (1), 90 subjects were selected for metabolic profiling by Rogers et.al.: 29 with SIRS, 30 with Sepsis, and 31 with sepsis-induced ARDS (4). Cases for the Rogers paper were selected for metabolic profiling based in part on IL-18 levels as part of the Dolinay et.al. study protocol (sepsis and SIRS patients with low IL-18 levels, ARDS with high IL-18 levels) (1). Rogers published metabolomic signatures of outcome in the RoCI and a second cohort (4). Of the 90 RoCI subjects with metabolite profiles completed in the Rogers study, 73 had enough plasma available to determine ND1 mtDNA whom were the subject of our current study (Table 1).

Blood samples were drawn and transferred into EDTA coated blood collection tubes within 24 hours from study inclusion and processed within 4 hours after venipuncture. Subsequently, plasma was fractionated, aliquoted and stored at -80°C (1). 150 μl aliquots were shipped on dry-ice to Metabolon, Inc. Following receipt, the frozen samples in the box were immediately stored at -80^o^C. Metabolites are noted by other investigators to be stable for at least two freeze thaw cycles (5).

All patients in the analytic cohort had at least 28-day follow up data available for review. 28 day mortality was determined on the complete cohort using hospital records and the Social Security Administration Death Master File (6). Race was either self-determined or designated by a patient representative/healthcare proxy. Presence of malignancy was determined by subject interview and medical record confirmation. Renal function, measured by glomerular filtration rate (GFR) was calculated with the Modification of Diet in Renal Disease (MDRD) equation from the baseline creatinine, age, gender, and race of cohort patients (33)*.* Classification of SIRS, sepsis, and ARDS, was determined by a consensus panel of ICU physicians using the Consensus Conference classifications (2, 3). Acute physiology and chronic health evaluation (APACHE) II score was determined at 24 hours post-ICU admission (7).

Preparation and Quantification of plasma ND1 mtDNA have been outlined at length (10). Briefly, in RoCI, blood samples were drawn and transferred into blood collection tubes within 24 hours from study inclusion and processed within 2 hours after venipuncture (11). Plasma used in this study was prepared by using EDTA-coated blood collection tubes. The plasma samples stored at –80°C were thawed on ice. The 100 µl of plasma was mixed with 100 µl of PBS, followed by brief vortex. The diluted plasma was centrifuged at 700g at 4°C for 5 minutes and the supernatant (190 µl) was carefully saved by avoiding touching any pellets and the bottom of the tubes. The obtained supernatant was further centrifuged at 18,000g at 4°C for 15 minutes and the resulting supernatant (170 µl) was carefully saved and processed for DNA isolation using DNeasy Blood and Tissue (Qiagen; Part Number 69504) according to the manufacture manual (12). For real-time quantitative polymerase chain reaction (qPCR) assay, the DNA solution was further diluted 10 times with nuclease-free deionized-distilled H_2_O.

DNA level in diluted samples was measured by SYBR® Green dye-based real-time qPCR assay PRISM 7300 sequence detection system (Applied Biosystems, Foster City, CA, USA). The references for primer sequences were as follows: Human NADH dehydrogenase 1 gene (ND1 mtDNA) (13, 14); Human β-globin (nuclear DNA)(15); Bacterial 16S ribosomal RNA (13). Plasmid DNA with cDNA sequences for human ND1 mtDNA was obtained from ORIGENE (SC101172, Rockville, MD) and plasmid DNA with cDNA sequences for human nuclear DNA was obtained from Sino Biological Inc. Concentrations were converted to copy number using the formula; mol/g × molecules/mol = molecules/g, via a DNA copy number calculator http://www.uri.edu/research/gsc/resources/cndna.html (16, 17). DNA solutions were diluted in 10-fold serial dilutions and used as standards.

All samples were analyzed in duplicate, and a no-template control was included in every analysis. ND1 mtDNA levels in all of the plasma analyses were expressed in copies per micro liter of plasma based on the following calculation (18): c = Q X VDNA/VPCR X 1/Vext. c is the concentration of DNA in plasma (copies/µl plasma); Q is the quantity (copies) of DNA determined by the sequence detector in a PCR; VDNA is the total volume of plasma DNA solution obtained after extraction; typically 200 µl per extraction; VPCR is the volume of plasma DNA solution used for PCR, typically 5 µl of 10 times-diluted plasma DNA solution; Vext is the volume of plasma extracted, typically 50-100 µl (10).

For the metabolite measurements, instrument variability was determined by calculating the median relative standard deviation (RSD) for the internal standards that were added to each sample prior to injection into the mass spectrometers. The median relative standard deviation for instrument variability was 5%. Overall process variability was determined by calculating the median RSD for all endogenous metabolites (i.e., non-instrument standards) present in 100% of the Client Matrix samples, which are technical replicates of pooled client samples. The median relative standard deviation for overall process variability was 11% (4).

**Supplemental Methods References**

1. Dolinay T, Kim YS, Howrylak J, et al. Inflammasome-regulated cytokines are critical mediators of acute lung injury. Am J Respir Crit Care Med 2012; 185(11):1225-1234

2. Levy MM, Fink MP, Marshall JC, et al. 2001 SCCM/ESICM/ACCP/ATS/SIS International Sepsis Definitions Conference. Crit Care Med 2003; 31(4):1250-1256

3. Bernard GR, Artigas A, Brigham KL, et al. The American-European Consensus Conference on ARDS. Definitions, mechanisms, relevant outcomes, and clinical trial coordination. Am J Respir Crit Care Med 1994; 149(3 Pt 1):818-824

4. Rogers AJ, McGeachie M, Baron RM, et al. Metabolomic derangements are associated with mortality in critically ill adult patients. PLoS One 2014; 9(1):e87538

5. Breier M, Wahl S, Prehn C, et al. Targeted metabolomics identifies reliable and stable metabolites in human serum and plasma samples. PLoS One 2014; 9(2):e89728

6. Sohn MW, Arnold N, Maynard C, et al. Accuracy and completeness of mortality data in the Department of Veterans Affairs. Popul Health Metr 2006; 4:2

7. Knaus WA, Draper EA, Wagner DP, et al. APACHE II: a severity of disease classification system. Crit Care Med 1985; 13(10):818-829

8. Robinson MK, Mogensen KM, Casey JD, et al. The relationship among obesity, nutritional status, and mortality in the critically ill*. Crit Care Med 2015; 43(1):87-100

9. Mogensen KM, Robinson MK, Casey JD, et al. Nutritional Status and Mortality in the Critically Ill. Critical care medicine 2015; 43(12):2605-2615

10. Nakahira K, Kyung SY, Rogers AJ, et al. Circulating mitochondrial DNA in patients in the ICU as a marker of mortality: derivation and validation. PLoS medicine 2013; 10(12):e1001577

11. Dolinay T, Kim YS, Howrylak J, et al. Inflammasome-Regulated Cytokines are Critical Mediators of Acute Lung Injury. Am J Respir Crit Care Med 2012;

12. Nakahira K, Haspel JA, Rathinam VA, et al. Autophagy proteins regulate innate immune responses by inhibiting the release of mitochondrial DNA mediated by the NALP3 inflammasome. Nat Immunol 2011; 12(3):222-230

13. Zhang Q, Raoof M, Chen Y, et al. Circulating mitochondrial DAMPs cause inflammatory responses to injury. Nature 2010; 464(7285):104-107

14. McGill MR, Sharpe MR, Williams CD, et al. The mechanism underlying acetaminophen-induced hepatotoxicity in humans and mice involves mitochondrial damage and nuclear DNA fragmentation. The Journal of clinical investigation 2012; 122(4):1574-1583

15. Moreira VG, Prieto B, Rodriguez JS, et al. Usefulness of cell-free plasma DNA, procalcitonin and C-reactive protein as markers of infection in febrile patients. Annals of clinical biochemistry 2010; 47(Pt 3):253-258

16. Nga TV, Karkey A, Dongol S, et al. The sensitivity of real-time PCR amplification targeting invasive Salmonella serovars in biological specimens. BMC infectious diseases 2010; 10:125

17. Zozaya-Hinchliffe M, Martin DH, Ferris MJ. Prevalence and abundance of uncultivated Megasphaera-like bacteria in the human vaginal environment. Applied and environmental microbiology 2008; 74(5):1656-1659

18. Chiu RW, Chan LY, Lam NY, et al. Quantitative analysis of circulating mitochondrial DNA in plasma. Clinical chemistry 2003; 49(5):719-726
