# Supplementary material for: Plasma mitochondrial DNA and metabolomic alterations in severe critical illness
Source: Crit Care. 2018 Dec 29;22:360. doi: 10.1186/s13054-018-2275-7 (PMC6310975; doi:10.1186/s13054-018-2275-7)
Supplement: Supplementary file 2 — Table S1. Metabolites identified via volcano plot with significant differences in patients with ND1 mtDNA ≥ 3200 copies/μl plasma relative to those with ND1 mtDNA < 3200 copies/μl plasma. Table S2. Linear regression of significant metabolites relative to every 100 ND1 mtDNA copies/μl plasma analyzed as a continuous exposure. Table S3. Metabolites identified by SAM with significant differences in patients with ND1 mtDNA ≥ 3200 copies/μl plasma relative to those with ND1 mtDNA < 3200 copies/μl plasma. (DOCX 33 kb) [file 13054_2018_2275_MOESM2_ESM.docx]

**Table S1.** Metabolites identified via Volcano plot with significant differences in patients with ND1 mtDNA ≥3,200 copies/µl plasma relative to those with ND1 mtDNA <3,200 copies/µl plasma.

| **Metabolite** | **Super Pathway** | **Sub Pathway** | **Fold Change** | **FDR- adjusted-p-value** |
| --- | --- | --- | --- | --- |
| 3-ureidopropionate | Amino acid | Alanine and aspartate metabolism | 6.66 | 0.01473 |
| Fucose | Carbohydrate | Aminosugars metabolism | 2.70 | 0.01602 |
| Taurocholate | Lipid | Bile acid metabolism | 2.62 | 0.01281 |
| 2-hydroxyglutarate | Lipid | Fatty acid, dicarboxylate | 5.74 | 0.01147 |
| Sucrose | Carbohydrate | Sucrose metabolism | 9.61 | 0.00839 |
| Gamma-glutamylphenylalanine | Peptide | Gamma-glutamyl | 3.45 | 0.01281 |
| Gamma-glutamyltyrosine | Peptide | Gamma-glutamyl | 2.87 | 0.0386 |
| scyllo-inositol | Lipid | Inositol metabolism | 2.50 | 0.04141 |
| N-6-trimethyllysine | Amino acid | Lysine metabolism | 2.95 | 0.0275 |
| Ribitol | Carbohydrate | Nucleotide sugars, pentose metabolism | 5.71 | 0.01602 |
| Xylitol | Carbohydrate | Nucleotide sugars, pentose metabolism | 2.50 | 0.0321 |
| Phenyllactate | Amino acid | Phenylalanine & tyrosine metabolism | 3.97 | 0.01147 |
| N-acetylphenylalanine | Amino acid | Phenylalanine & tyrosine metabolism | 2.99 | 0.03557 |
| 3-(4-hydroxyphenyl)lactate | Amino acid | Phenylalanine & tyrosine metabolism | 2.78 | 0.03634 |
| 5-methylthioadenosine | Amino acid | Polyamine metabolism | 2.12 | 0.03565 |
| N2,N2-dimethylguanosine | Nucleotide | Purine metabolism, guanine containing | 2.54 | 0.03618 |
| N4-acetylcytidine | Nucleotide | Pyrimidine metabolism, cytidine containing | 2.82 | 0.01602 |
| **Propionylcarnitine (C3)** | Lipid | Short Chain Acyl Carnitine | 4.06 | 0.01473 |
| **Isobutyrylcarnitine (C4)** | Lipid | Short Chain Acyl Carnitine | 4.49 | 0.00577 |
| **Butyrylcarnitine (C4)** | Lipid | Short Chain Acyl Carnitine | 2.53 | 0.01937 |
| **Isovalerylcarnitine (C5)** | Lipid | Short Chain Acyl Carnitine | 4.01 | 0.00623 |
| **2-methylbutyroylcarnitine (C5)** | Lipid | Short Chain Acyl Carnitine | 4.30 | 0.00067 |
| **Hydroxyisovaleroyl carnitine (C5)** | Lipid | Short Chain Acyl Carnitine | 2.32 | 0.01722 |
| **Tiglyl carnitine (C5)** | Lipid | Short Chain Acyl Carnitine | 2.12 | 0.00693 |
| **Hexanoylcarnitine (C6)** | Lipid | Short Chain Acyl Carnitine | 2.65 | 0.02097 |
| Kynurenine | Amino acid | Tryptophan metabolism | 7.15 | 0.00839 |
| Urea | Amino acid | Urea cycle; arginine, proline, metabolism | 2.02 | 0.00463 |
| 3-hydroxyisobutyrate | Amino acid | Valine, leucine and isoleucine metabolism | 3.27 | 0.01473 |
| Serine | Amino acid | Glycine, serine and threonine metabolism | 0.36 | 0.04141 |
| **Palmitoylcarnitine (C16)** | Lipid | Long Chain acylcarnitine | 0.48 | 0.01267 |
| **Oleoylcarnitine (C18)** | Lipid | Long Chain acylcarnitine | 0.41 | 0.01473 |
| **1-eicosatrienoylglycerophosphocholine** | Lipid | Lysolipid | 0.45 | 0.01473 |
| **1-pentadecanoylglycerophosphocholine** | Lipid | Lysolipid | 0.44 | 0.04708 |
| **2-linoleoylglycerophosphocholine** | Lipid | Lysolipid | 0.41 | 0.00367 |
| **1-docosahexaenoylglycerophosphocholine** | Lipid | Lysolipid | 0.38 | 0.00839 |
| **1-palmitoylglycerophosphocholine** | Lipid | Lysolipid | 0.37 | 0.00067 |
| **1-myristoylglycerophosphocholine** | Lipid | Lysolipid | 0.37 | 0.00794 |
| **1-palmitoleoylglycerophosphocholine** | Lipid | Lysolipid | 0.34 | 0.00067 |
| **1-docosapentaenoylglycerophosphocholine** | Lipid | Lysolipid | 0.33 | 0.02036 |
| **1-linoleoylglycerophosphocholine** | Lipid | Lysolipid | 0.32 | 0.0016 |
| **2-palmitoylglycerophosphocholine** | Lipid | Lysolipid | 0.32 | 0.00067 |
| **1-stearoylglycerophosphocholine** | Lipid | Lysolipid | 0.31 | 0.0036 |
| **1-heptadecanoylglycerophosphocholine** | Lipid | Lysolipid | 0.31 | 0.00196 |
| **1-oleoylglycerophosphocholine** | Lipid | Lysolipid | 0.30 | 0.00067 |
| **2-stearoylglycerophosphocholine** | Lipid | Lysolipid | 0.29 | 0.00661 |
| **1-arachidonoylglycerophosphocholine** | Lipid | Lysolipid | 0.28 | 0.00173 |
| 1-linoleoylglycerophosphoethanolamine | Lipid | Lysolipid | 0.32 | 0.01985 |
| 1-arachidonoylglycerophosphoethanolamine | Lipid | Lysolipid | 0.25 | 0.00693 |
| 2-arachidonoylglycerophosphoethanolamine | Lipid | Lysolipid | 0.23 | 0.01281 |

Note: Bold text highlight glycerophosphocholine and acylcarnitine metabolites.

| **Metabolite** | **Super Pathway** | **Sub Pathway** | **Beta Coefficient** | **P-value** |
| --- | --- | --- | --- | --- |
| **Butyrylcarnitine (C4)** | Lipid | Short Chain Acyl Carnitine | 1.50E-03 | 0.022 |
| **Propionylcarnitine (C3)** | Lipid | Short Chain Acyl Carnitine | 1.53E-03 | 0.025 |
| **Isovalerylcarnitine (C5)** | Lipid | Short Chain Acyl Carnitine | 2.54E-03 | 0.0034 |
| **2-methylbutyroylcarnitine (C5)** | Lipid | Short Chain Acyl Carnitine | 1.91E-03 | 0.012 |
| **1-stearoylglycerophosphocholine** | Lipid | Lysolipid | -2.90E-03 | 0.014 |
| **2-stearoylglycerophosphocholine** | Lipid | Lysolipid | -2.70E-03 | 0.015 |
| **1-palmitoleoylglycerophosphocholine** | Lipid | Lysolipid | -2.90E-03 | 0.015 |
| **1-arachidonoylglycerophosphoethanolamine** | Lipid | Lysolipid | -1.90E-03 | 0.016 |
| **1-myristoylglycerophosphocholine** | Lipid | Lysolipid | -2.50E-03 | 0.028 |
| **1-linoleoylglycerophosphocholine** | Lipid | Lysolipid | -2.60E-03 | 0.031 |
| 2-hydroxyglutarate | Lipid | Fatty acid, dicarboxylate | 2.07E-03 | 0.0069 |
| Choline | Lipid | Glycerolipid metabolism | 1.08E-03 | 0.011 |
| Glycerate | Carbohydrate | Glycolysis, gluconeogenesis, pyruvate metabolism | 1.19E-03 | 0.012 |
| Heme | Cofactors and vitamins | Hemoglobin and porphyrin metabolism | 2.87E-03 | 0.0035 |
| Urobilinogen | Cofactors and vitamins | Hemoglobin and porphyrin metabolism | 4.12E-03 | 0.0056 |
| 1-methylimidazoleacetate | Amino acid | Histidine metabolism | 2.25E-03 | 0.024 |
| phenyllactate (PLA) | Amino acid | Phenylalanine & tyrosine metabolism | 2.60E-03 | 0.015 |
| 3-(4-hydroxyphenyl)lactate | Amino acid | Phenylalanine & tyrosine metabolism | 2.40E-03 | 0.017 |
| Hypoxanthine | Nucleotide | Purine metabolism | -2.10E-03 | 0.0045 |
| Pregnenolone sulfate | Lipid | Sterol/Steroid | 3.74E-03 | 0.00031 |
| Pregn steroid monosulfate | Lipid | Sterol/Steroid | 3.47E-03 | 0.0031 |
| Estrone 3-sulfate | Lipid | Sterol/Steroid | 3.47E-03 | 0.0074 |
| Alpha-hydroxyisovalerate | Amino acid | Valine, leucine and isoleucine metabolism | 2.07E-03 | 0.02 |
| 4-acetamidophenol | Xenobiotics | Drug | 7.01E-03 | 0.00051 |
| 3-(cystein-S-yl)acetaminophen | Xenobiotics | Drug | 5.14E-03 | 0.023 |
| 2-methoxyacetaminophen sulfate | Xenobiotics | Drug | 4.67E-03 | 0.029 |

**Table S2** Linear regression of significant metabolites relative to every 100 ND1 mtDNA copies/µl plasma analyzed as continuous.

Note: Estimates adjusted for age, gender, race and APACHE II score

**Table S3.** Metabolites identified by SAM with significant differences in patients with ND1 mtDNA ≥3,200 copies/µl plasma relative to those with ND1 mtDNA <3,200 copies/µl plasma.

| **Metabolite** | **Super Pathway** | **Sub Pathway** | **d-value** | **Raw p-value** | **q-value** |
| --- | --- | --- | --- | --- | --- |
| 3-ureidopropionate | Amino acid | Alanine and aspartate metabolism | 3.02 | 0.0041 | 0.011 |
| Fucose | Carbohydrate | Aminosugars metabolism | 3.54 | 0.00070 | 0.0036 |
| Taurocholate | Lipid | Bile acid metabolism | 3.31 | 0.0014 | 0.0059 |
| Taurochenodeoxycholate | Lipid | Bile acid metabolism | 2.86 | 0.0066 | 0.016 |
| 2-hydroxyphenylacetate | Xenobiotics | Chemical | 2.83 | 0.0072 | 0.016 |
| 2-hydroxyglutarate | Lipid | Fatty acid, dicarboxylate | 3.16 | 0.0023 | 0.0076 |
| Sucrose | Carbohydrate | Sucrose metabolism | 3.68 | 0.00050 | 0.0031 |
| Gamma-glutamylphenylalanine | Peptide | gamma-glutamyl | 3.39 | 0.0012 | 0.0055 |
| N-acetylserine | Amino acid | Glycine, serine and threonine metabolism | 3.05 | 0.0037 | 0.011 |
| 1-methylimidazoleacetate | Amino acid | Histidine metabolism | 3.09 | 0.0032 | 0.0097 |
| 1,2-propanediol | Lipid | Ketone bodies | 2.91 | 0.0056 | 0.014 |
| N-6-trimethyllysine | Amino acid | Lysine metabolism | 2.96 | 0.0048 | 0.013 |
| Xylitol | Carbohydrate | Nucleotide sugars, pentose metabolism | 3.27 | 0.0017 | 0.0068 |
| Phenyllactate | Amino acid | Phenylalanine & tyrosine metabolism | 3.66 | 0.00060 | 0.0031 |
| 3-(4-hydroxyphenyl)lactate | Amino acid | Phenylalanine & tyrosine metabolism | 3.21 | 0.0020 | 0.0073 |
| N-acetylphenylalanine | Amino acid | Phenylalanine & tyrosine metabolism | 2.87 | 0.0063 | 0.016 |
| N2,N2-dimethylguanosine | Nucleotide | Purine metabolism, guanine containing | 2.95 | 0.0049 | 0.013 |
| N4-acetylcytidine | Nucleotide | Pyrimidine metabolism, cytidine containing | 3.17 | 0.0023 | 0.0076 |
| **Propionylcarnitine (C3)** | Lipid | Short Chain Acyl Carnitine | 4.20 | 0.20 | 0.0015 |
| **Isobutyrylcarnitine (C4)** | Lipid | Short Chain Acyl Carnitine | 3.92 | 0.27 | 0.0023 |
| **Butyrylcarnitine (C4)** | Lipid | Short Chain Acyl Carnitine | 3.59 | 0.20 | 0.0036 |
| **Isovalerylcarnitine (C5)** | Lipid | Short Chain Acyl Carnitine | 4.17 | 0.26 | 0.0015 |
| **2-methylbutyroylcarnitine (C5)** | Lipid | Short Chain Acyl Carnitine | 4.42 | 0.23 | 0.00059 |
| **Hydroxyisovaleroyl carnitine (C5)** | Lipid | Short Chain Acyl Carnitine | 2.86 | 0.29 | 0.016 |
| **Tiglyl carnitine (C5)** | Lipid | Short Chain Acyl Carnitine | 3.03 | 0.23 | 0.011 |
| **Hexanoylcarnitine (C6)** | Lipid | Short Chain Acyl Carnitine | 3.71 | 0.22 | 0.0031 |
| Kynurenine | Amino acid | Tryptophan metabolism | 4.01 | 0.00030 | 0.0023 |
| Kynurenate | Amino acid | Tryptophan metabolism | 2.85 | 0.0068 | 0.016 |
| 3-hydroxyisobutyrate | Amino acid | Valine, leucine and isoleucine metabolism | 3.71 | 0.00050 | 0.0031 |
| 4-vinylphenol sulfate | Xenobiotics | Benzoate metabolism | -2.94 | 0.0051 | 0.013 |
| 13-HODE + 9-HODE | Lipid | Fatty acid, monohydroxy | -3.64 | 0.00060 | 0.0031 |
| N-acetylglycine | Amino acid | Glycine, serine and threonine metabolism | -3.10 | 0.0032 | 0.0097 |
| Citrate | Energy | Krebs cycle | -3.16 | 0.0023 | 0.0076 |
| **Oleoylcarnitine (C18)** | Lipid | Long Chain acylcarnitine | -3.18 | 0.0022 | 0.0076 |
| **Palmitoylcarnitine (C16)** | Lipid | Long Chain acylcarnitine | -3.29 | 0.0017 | 0.0068 |
| 1-palmitoylplasmenylethanolamine | Lipid | Lysolipid | -3.05 | 0.0036 | 0.011 |
| 1-oleoylglycerophosphoethanolamine | Lipid | Lysolipid | -3.15 | 0.0024 | 0.0076 |
| 2-linoleoylglycerophosphocholine | Lipid | Lysolipid | -3.21 | 0.0020 | 0.0073 |
| **2-oleoylglycerophosphocholine** | Lipid | Lysolipid | -3.23 | 0.0019 | 0.0073 |
| 1-linoleoylglycerophosphoethanolamine | Lipid | Lysolipid | -3.36 | 0.0013 | 0.0057 |
| 2-arachidonoylglycerophosphoethanolamine | Lipid | Lysolipid | -3.40 | 0.0012 | 0.0055 |
| **1-docosapentaenoylglycerophosphocholine** | Lipid | Lysolipid | -3.42 | 0.0011 | 0.0052 |
| **1-eicosatrienoylglycerophosphocholine** | Lipid | Lysolipid | -3.87 | 0.00032 | 0.0023 |
| **1-docosahexaenoyl****glycerophosphocholine** | Lipid | Lysolipid | -4.01 | 0.00029 | 0.0023 |
| **1-myristoylglycerophosphocholine** | Lipid | Lysolipid | -4.20 | 0.00016 | 0.0015 |
| **1-stearoylglycerophosphocholine** | Lipid | Lysolipid | -4.21 | 0.00016 | 0.0015 |
| **1-palmitoleoylglycerophosphocholine** | Lipid | Lysolipid | -4.24 | 0.00013 | 0.0015 |
| **1-heptadecanoylglycerophosphocholine** | Lipid | Lysolipid | -4.24 | 0.00013 | 0.0015 |
| 1-arachidonoylglycerophosphoethanolamine | Lipid | Lysolipid | -4.28 | 0.00010 | 0.0015 |

Note: d-value analogous to Student’s t-statistic, q-value is the upper limit for the False Discovery Rate (FDR). Bold text highlight glycerophosphocholine and acylcarnitine metabolites. Metabolites that are significantly differentially concentrated with q value<0.01 (upper limit for the FDR) in ND1 mtDNA groups are shown.

**Supplemental Table 4.** Differentially accumulated metabolites identified by Variable Importance in the Projection values >1 in patients with ND1 mtDNA ≥3,200 copies/µl plasma relative to those with ND1 mtDNA <3,200 copies/µl plasma.

| **Metabolite** | **Super Pathway** | **Sub Pathway** |
| --- | --- | --- |
| **Butyrylcarnitine (C4)** | Lipid | Short Chain Acyl Carnitine |
| **Methylglutaroylcarnitine (C6)** | Lipid | Short Chain Acyl Carnitine |
| **Isovalerylcarnitine (C5)** | Lipid | Short Chain Acyl Carnitine |
| **Glycerophosphorylcholine** | Lipid | Glycerolipid metabolism |
| **1-arachidonoyl****glycerophosphocholine** | Lipid | Lysolipid |
| **1-palmitoleoylglycerophosphocholine** | Lipid | Lysolipid |
| **1-stearoylglycerophosphocholine** | Lipid | Lysolipid |
| **1-myristoylglycerophosphocholine** | Lipid | Lysolipid |
| **1-docosapentaenoylglycerophosphocholine** | Lipid | Lysolipid |
| **2-oleoylglycerophosphocholine** | Lipid | Lysolipid |
| 1-arachidonoylglycerophosphoethanolamine | Lipid | Lysolipid |
| 1-palmitoylglycerophosphoethanolamine | Lipid | Lysolipid |
| 3-ureidopropionate | Amino acid | Alanine and aspartate metabolism |
| N-acetyl-beta-alanine | Amino acid | Alanine and aspartate metabolism |
| Fucose | Carbohydrate | Aminosugars metabolism |
| 4-hydroxyhippurate | Xenobiotics | Benzoate metabolism |
| 3-hydroxyhippurate | Xenobiotics | Benzoate metabolism |
| Cysteine | Amino acid | Cysteine, methionine, SAM, taurine metabolism |
| Sucrose | Carbohydrate | Sucrose metabolism |
| Sorbitol | Carbohydrate | Sucrose metabolism |
| Glutamate | Amino acid | Glutamate metabolism |
| Glucuronate | Carbohydrate | Glycolysis, gluconeogenesis, pyruvate metabolism |
| 4-acetamidobutanoate | Amino acid | Guanidino and acetamido metabolism |
| trans-urocanate | Amino acid | Histidine metabolism |
| scyllo-inositol | Lipid | Inositol metabolism |
| Fumarate | Energy | Krebs cycle |
| Threitol | Carbohydrate | Nucleotide sugars, pentose metabolism |
| 3-(4-hydroxyphenyl)lactate | Amino acid | Phenylalanine & tyrosine metabolism |
| N2,N2-dimethylguanosine | Nucleotide | Purine metabolism, guanine containing |
| 7-methylguanine | Nucleotide | Purine metabolism, guanine containing |
| 5alpha-pregnan-3alpha,20beta-diol disulfate 1 | Lipid | Sterol/Steroid |
| Cortisol | Lipid | Sterol/Steroid |
| Kynurenate | Amino acid | Tryptophan metabolism |
| 3-hydroxy-2-ethylpropionate | Amino acid | Valine, leucine and isoleucine metabolism |

Note: Bold text highlight glycerophosphocholine and acylcarnitine metabolites.

**Supplemental Table 5.** OPLS-DA loadings [(p(1)] and the correlation p(corr)[1] of significant metabolites towards class segregation between ND1 mtDNA groups as determined by S-plot.

| **Metabolite** | p[1] | p(corr)[1] | **Super Pathway** | **Sub Pathway** |
| --- | --- | --- | --- | --- |
| **1-arachidonoylglycerophosphocholine** | -6.8485 | -0.73449 | Lipid | Lysolipid |
| **2-palmitoylglycerophosphocholine** | -5.8988 | -0.70657 | Lipid | Lysolipid |
| **1-palmitoylglycerophosphocholine** | -6.0189 | -0.68952 | Lipid | Lysolipid |
| **1-oleoylglycerophosphocholine** | -6.0232 | -0.67092 | Lipid | Lysolipid |
| **1-docosahexaenoylglycerophosphocholine** | -5.625 | -0.66445 | Lipid | Lysolipid |
| **1-linoleoylglycerophosphocholine** | -6.3701 | -0.6609 | Lipid | Lysolipid |
| **1-heptadecanoylglycerophosphocholine** | -5.053 | -0.64927 | Lipid | Lysolipid |
| 1-arachidonoylglycerophosphoethanolamine | -4.0174 | -0.64846 | Lipid | Lysolipid |
| **1-stearoylglycerophosphocholine** | -5.9516 | -0.64047 | Lipid | Lysolipid |
| **Palmitoylcarnitine (C16)** | -4.4402 | -0.63869 | Lipid | Long Chain acylcarnitine |
| **1-eicosatrienoylglycerophosphocholine** | -5.4166 | -0.63408 | Lipid | Lysolipid |
| **2-stearoylglycerophosphocholine** | -5.4475 | -0.62733 | Lipid | Lysolipid |
| **Oleoylcarnitine (C18)** | -3.8105 | -0.61019 | Lipid | Long Chain acylcarnitine |
| **1-myristoylglycerophosphocholine** | -5.3336 | -0.60219 | Lipid | Lysolipid |
| **1-palmitoleoylglycerophosphocholine** | -5.6489 | -0.59812 | Lipid | Lysolipid |
| **1-docosapentaenoylglycerophosphocholine** | -3.3434 | -0.58227 | Lipid | Lysolipid |
| **2-linoleoylglycerophosphocholine** | -3.802 | -0.5718 | Lipid | Lysolipid |
| 13-HODE + 9-HODE | -3.2498 | -0.55443 | Lipid | Fatty acid, monohydroxy |
| **2-oleoylglycerophosphocholine** | -3.7284 | -0.5379 | Lipid | Lysolipid |
| 1-linoleoylglycerophosphoethanolamine | -3.3647 | -0.52595 | Lipid | Lysolipid |
| Dihomo-linoleate (20:2n6) | -2.3642 | -0.52386 | Lipid | Long chain fatty acid |
| Dihomo-linolenate (20:3n3 or n6) | -1.8196 | -0.52295 | Lipid | Essential fatty acid |
| 1-palmitoylplasmenylethanolamine | -2.9554 | -0.51565 | Lipid | Lysolipid |
| **1-pentadecanoylglycerophosphocholine** | -3.3117 | -0.51374 | Lipid | Lysolipid |
| Citrate | -2.0756 | -0.50586 | Energy | Krebs cycle |
| **Stearoylcarnitine (C18)** | -2.7188 | -0.48279 | Lipid | Long Chain acylcarnitine |
| 10-heptadecenoate (17:1n7) | -2.5453 | -0.47396 | Lipid | Long chain fatty acid |
| cis-vaccenate (18:1n7) | -1.8605 | -0.466 | Lipid | Long chain fatty acid |
| Margarate (17:0) | -1.8004 | -0.45903 | Lipid | Long chain fatty acid |
| Myristate (14:0) | -1.6031 | -0.45573 | Lipid | Long chain fatty acid |
| 1-oleoylglycerophosphoethanolamine | -2.759 | -0.45419 | Lipid | Lysolipid |
| 2-arachidonoylglycerophosphoethanolamine | -2.2727 | -0.4494 | Lipid | Lysolipid |
| Palmitoleate (16:1n7) | -2.5468 | -0.44825 | Lipid | Long chain fatty acid |
| 10-nonadecenoate (19:1n9) | -2.4357 | -0.44282 | Lipid | Long chain fatty acid |
| 4-methyl-2-oxopentanoate | -1.8864 | -0.44229 | Amino acid | Valine, leucine and isoleucine metabolism |
| 3-methylhistidine | -4.2421 | -0.43912 | Amino acid | Histidine metabolism |
| Myristoleate (14:1n5) | -2.7276 | -0.43822 | Lipid | Long chain fatty acid |
| 3-methyl-2-oxovalerate | -1.6154 | -0.43804 | Amino acid | Valine, leucine and isoleucine metabolism |
| Docosapentaenoate (n6 DPA; 22:5n6) | -2.5951 | -0.43592 | Lipid | Essential fatty acid |
| Serotonin (5HT) | -2.3681 | -0.4313 | Amino acid | Tryptophan metabolism |
| 4-vinylphenol sulfate | -4.2687 | -0.41251 | Xenobiotics | Benzoate metabolism |
| 16-hydroxypalmitate | -1.6548 | -0.41228 | Lipid | Fatty acid, monohydroxy |
| **1-eicosadienoylglycerophosphocholine** | -3.0534 | -0.41026 | Lipid | Lysolipid |
| N-acetylphenylalanine | 2.4297 | 0.41125 | Amino acid | Phenylalanine & tyrosine metabolism |
| **Butyrylcarnitine (C4)** | 2.1027 | 0.41596 | Lipid | Short Chain Acyl Carnitine |
| **Tiglyl carnitine (C5)** | 2.442 | 0.42329 | Lipid | Short Chain Acyl Carnitine |
| 2-hydroxyphenylacetate | 3.1958 | 0.43952 | Xenobiotics | Chemical |
| **Hydroxyisovaleroyl carnitine (C5)** | 3.1468 | 0.44324 | Lipid | Short Chain Acyl Carnitine |
| **Hexanoylcarnitine (C6)** | 2.6476 | 0.46096 | Lipid | Short Chain Acyl Carnitine |
| Glucarate (saccharate) | 7.3776 | 0.47033 | Cofactors and vitamins | Ascorbate and aldarate metabolism |
| Glucuronate | 4.9564 | 0.4785 | Carbohydrate | Glycolysis, gluconeogenesis, pyruvate metabolism |
| 3-ureidopropionate | 2.9731 | 0.48628 | Amino acid | Alanine and aspartate metabolism |
| N-6-trimethyllysine | 2.5824 | 0.48966 | Amino acid | Lysine metabolism |
| Glycocholate | 5.8411 | 0.49121 | Lipid | Bile acid metabolism |
| Taurochenodeoxycholate | 6.4185 | 0.49409 | Lipid | Bile acid metabolism |
| N4-acetylcytidine | 2.0347 | 0.49556 | Nucleotide | Pyrimidine metabolism, cytidine containing |
| 1,2-propanediol | 8.104 | 0.49625 | Lipid | Ketone bodies |
| 5-methylthioadenosine (MTA) | 2.5763 | 0.50196 | Amino acid | Polyamine metabolism |
| Phenyllactate | 4.1308 | 0.50648 | Amino acid | Phenylalanine & tyrosine metabolism |
| **Isobutyrylcarnitine (C4)** | 3.5018 | 0.50811 | Lipid | Short Chain Acyl Carnitine |
| Gamma-glutamylphenylalanine | 2.7466 | 0.51995 | Peptide | gamma-glutamyl |
| **Isovalerylcarnitine (C5)** | 3.5023 | 0.52107 | Lipid | Short Chain Acyl Carnitine |
| Fucose | 3.337 | 0.52565 | Carbohydrate | Aminosugars metabolism |
| Taurocholate | 7.5194 | 0.53731 | Lipid | Bile acid metabolism |
| **Propionylcarnitine (C3)** | 2.8018 | 0.54233 | Lipid | Short Chain Acyl Carnitine |
| **2-methylbutyroylcarnitine (C5)** | 3.3462 | 0.5499 | Lipid | Short Chain Acyl Carnitine |
| Sucrose | 11.008 | 0.55829 | Carbohydrate | Sucrose metabolism |
| Kynurenine | 3.1221 | 0.56444 | Amino acid | Tryptophan metabolism |
| 1-methylimidazoleacetate | 4.4265 | 0.57865 | Amino acid | Histidine metabolism |
| 3-hydroxyisobutyrate | 2.9972 | 0.57916 | Amino acid | Valine, leucine and isoleucine metabolism |
| Xylitol | 4.7368 | 0.61663 | Carbohydrate | Nucleotide sugars, pentose metabolism |

Note: OPLS-DA: orthogonal partial least squares-discriminant analysis; p[1]: OPLS-DA loadings; p(corr)[1]: correlation coefficient. p(corr)[1] of ±0.410 was adopted as a cutoff value to select the variables that are most correlated with the OPLS-DA discriminant scores. Bold text highlight glycerophosphocholine and acylcarnitine metabolites.
