# Supplementary material for: Plasma mitochondrial DNA and metabolomic alterations in severe critical illness
Source: Crit Care. 2018 Dec 29;22:360. doi: 10.1186/s13054-018-2275-7 (PMC6310975; doi:10.1186/s13054-018-2275-7)

**Figure S1. Acylcarnitine Association Plot.** Logistic regression results of 13 acylcarnitine esters in 73 patients. Each dot is a single acylcarnitine ester detected. Color indicates the relative acylcarnitine ester association with ND1 mtDNA ≥3,200 copies/µl plasma (red increased, blue decreased) after adjustment for age, gender, race, malignancy and APACHE II. Y axis is (-log_10_(p) value). X axis is acylcarnitine chain length C3 to C18.


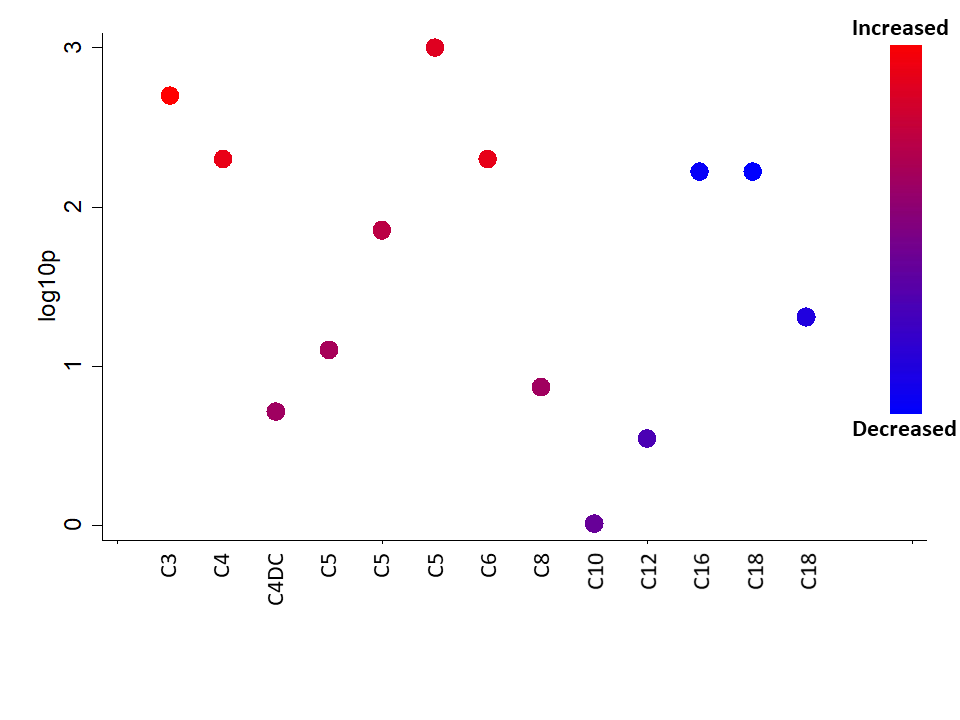

Supplement: Supplementary file 3 — Figure S1. Acylcarnitine association plot. Logistic regression results of 13 acylcarnitine esters in 73 patients. Each dot is a single acylcarnitine ester detected. Color indicates the relative acylcarnitine ester association with ND1 mtDNA ≥ 3200 copies/μl plasma (red increased, blue decreased) after adjustment for age, sex, race, malignancy, and APACHE II score. y-Axis is (−log10(p) value). x-Axis is acylcarnitine chain length C3 to C18. (DOCX 38 kb) [file 13054_2018_2275_MOESM3_ESM.docx]
